# Ultrafast response of monolayer molybdenum disulfide photodetectors

**DOI:** 10.1038/ncomms9831

**Published:** 2015-11-17

**Authors:** Haining Wang, Changjian Zhang, Weimin Chan, Sandip Tiwari, Farhan Rana

**Affiliations:** 1 School of Electrical and Computer Engineering, Cornell University, Ithaca 14853, New York, USA

## Abstract

The strong light emission and absorption exhibited by single atomic layer transitional metal dichalcogenides in the visible to near-infrared wavelength range make them attractive for optoelectronic applications. In this work, using two-pulse photovoltage correlation technique, we show that monolayer molybdenum disulfide photodetector can have intrinsic response times as short as 3 ps implying photodetection bandwidths as wide as 300 GHz. The fast photodetector response is a result of the short electron–hole and exciton lifetimes in this material. Recombination of photoexcited carriers in most two-dimensional metal dichalcogenides is dominated by nonradiative processes, most notable among which is Auger scattering. The fast response time, and the ease of fabrication of these devices, make them interesting for low-cost ultrafast optical communication links.

## Abstract (web summary)

Two-dimensional transition-metal dichalcogenides are useful for optoelectronic applications, but the ultimate limit on the speed of photodetector operation is unknown. Here, the authors show that the optical response time of monolayer molybdenum disulfide can be as short as three picoseconds.

## Graphical abstract

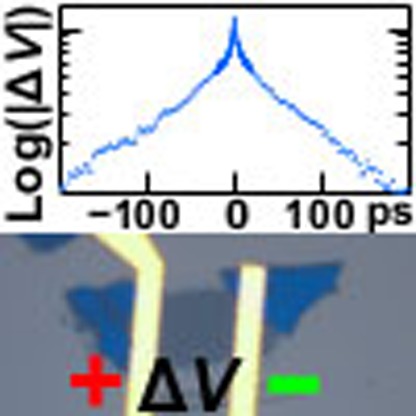

Two-dimensional (2D) transition metal dichalcogenides (TMDs) have emerged as interesting materials for low-cost optoelectronic devices, including photodetectors, light-emitting diodes, and, more recently, lasers^1^,^2^,^3^,^4^,^5^,^6^,^7^,^8^,^9^,^10^,^11^. In the case of photodetectors, the response time and the quantum efficiency are two important figures of merit. The intrinsic response time of TMD photodetectors and the ultimate limits on the speed of operation are unknown. The reported quantum efficiencies of TMD materials and devices are typically in the 0.0001–0.01 range^5^,^6^,^7^,^8^,^9^,^12^,^13^,^14^,^15^, indicating that most of the electrically injected or optically generated electrons and holes recombine nonradiatively. Understanding the nonradiative carrier recombination mechanisms, as well as the associated timescales, is therefore important. Previously, ultrafast optical/THz pump–probe as well as ultrafast photoluminescence techniques have been used, by the authors and others, to study the ultrafast carrier dynamics in metal dichalcogenides and in molybdenum disulfide (MoS_2_) in particular^12^,^16^,^17^,^18^,^19^,^20^,^21^,^22^. In these measurements, free-carrier recombination dynamics, exciton formation and recombination dynamics, refractive index changes, optical/THz intraband and interband conductivity changes, as well as the dynamics associated with carriers trapped in optically active midgap defects are all expected to play a role to varying degrees and, consequently, the results have been difficult to interpret and reconcile.

In this letter, we present experimental results on ultrafast two-pulse photovoltage correlation (TPPC) measurements on monolayer MoS_2_ metal-semiconductor photodetectors. In TPPC measurements, a photodetector is excited with two identical optical pulses separated by a time delay and the integrated detector photoresponse (either photovoltage or photocurrent response) is recorded as a function of the time delay. TPPC thus uses the photodetector to perform an optical correlation measurement. The nonlinearity of the photoresponse with respect to the optical pulse energy enables one to determine ultrafast intrinsic temporal response of the detector with sub-picosecond resolution^23^,^24^,^25^,^26^,^27^. Our measurements show that the photovoltage is suppressed when the two optical pulses arrive together indicating a saturation of the photoresponse. As the time delay between the two pulses is increased from zero, the photovoltage recovers, and the recovery, as a function of the time delay, exhibits two distinct timescales: (i) a fast timescale of the order of 3–5 ps and (ii) a slow timescale of ∼80–110 ps. These two timescales are found to be largely independent of the temperature, exhibits only a mild dependence on the pump fluence, and varies a little from sample to sample. Between 50 and 75% of the photovoltage correlation response recovers on the fast timescale implying that ultrafast TMD photodetectors with (8 dB) current modulation bandwidths in the 200–300 GHz range are possible. The fast response speed is a result of the short lifetime of the photoexcited carriers. Since TPPC measures the photovoltage (or photocurrent), this technique is sensitive only to the total photoexcited carrier population, including both bound (excitons) and free carriers, that contributes to the photoresponse. TPPC therefore also offers important and unique insights into the carrier recombination dynamics. The temperature and pump fluence dependence of our TPPC results are consistent with defect-assisted recombination as being the dominant mechanism, in which the the photoexcited electrons and holes, both free and bound (excitons), are captured by defects via Auger scattering^28^. Strong Coulomb interactions in 2D TMDs, including the correlations in the positions of free and bound electrons and holes arising from the attractive interactions, result in large carrier capture rates by defects via Auger scattering^28^. Our results underscore the trade-off between speed and quantum efficiency in TMD photodetectors.

## Results

### Two-pulse photovoltage correlation technique

**Figure 1:**
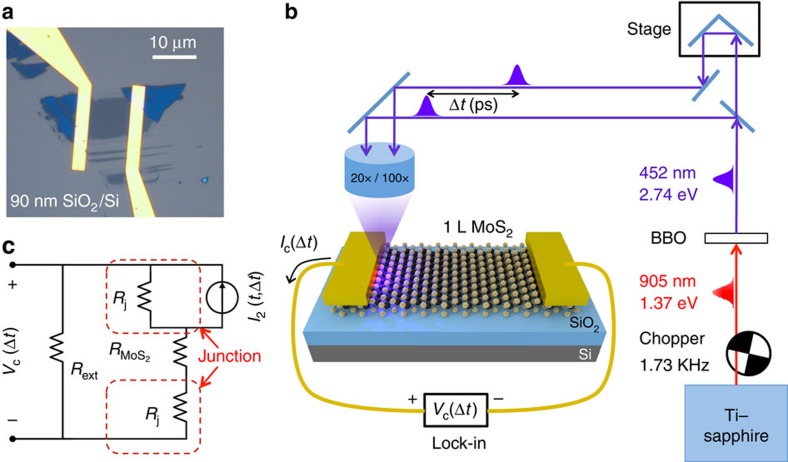
TPPC experiment and circuit model of metal-MoS_2_ photodetector. (**a**) Optical micrograph of a fabricated back-gated monolayer metal-MoS_2_ photodetector on SiO_2_/Si substrate is shown. (**b**) A schematic of the two-pulse photovoltage correlation (TPPC) experiment is shown. Two time-delayed 452 nm optical pulses, both obtained via upconversion from a single Ti:Sapphire laser, are focused at one of the metal-semiconductor Schottky junctions. The generated d.c. photovoltage is recorded as a function of the time delay between the pulses. A lock-in detection scheme is used to improve the signal-to-noise ratio. The arrow indicates the positive direction of the photocurrent (and the sign of the measured photovoltage) form the illuminated metal contact. (**c**) A low-frequency circuit model of the device and measurement. The current source *I*_2_(*t*, Δ*t*) represents the short circuit current response of the junction in response to two optical pulses separated by time Δ*t*. *R*_j_ is the resistance of the metal-MoS_2_ junction. 
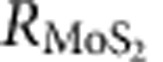
 is the resistance of the MoS_2_ layer. *R*_ext_ is the external circuit resistance (including the ∼10 MΩ input resistance of the measurement instrument).

Microscope image of a monolayer metal-MoS_2_ photodetector is shown in Fig. 1a, and the schematic in Fig. 1b depicts the setup for a TPPC experiment. A ∼80-fs, 905-nm (1.37 eV) centre wavelength, optical pulse from a ∼83-MHz repetition rate Ti–Sapphire laser is frequency doubled to 452 nm (2.74 eV, ∼150 fs) by a beta-BaB_2_O_4_ crystal, then mechanically chopped at 1.73 KHz and then split into two pulses by a 50/50 beam splitter. The time delay Δ*t* between these two pulses is controlled by a linear translation stage. The resulting voltage across the photodetector is measured as a function of the time delay between the pulses using a lock-in amplifier with a 10 MΩ input resistance. In experiments, the maximum photoresponse was obtained when the light was focused on the sample near one of the metal contacts of the device, and the photoresponse decayed rapidly as the centre of the focus spot was moved more than half a micron away from the metal contact. The direction of the d.c. photocurrent, and the resulting sign of the measured d.c. photovoltage are shown in Fig. 1b, and were determined without using the lock-in. Photovoltage was always positive at the contact near which the light was focused. Figure 1c shows a low-frequency circuit model of the device and the measurement. The circuit model shown can be derived from a high-frequency circuit model (Supplementary Fig. 2 and Supplementary Note 3). If the time-dependent short circuit current response of the illuminated junction to a single optical pulse is *I*_1_(*t*), and to two optical pulses separated by time Δ*t* is *I*_2_(*t*, Δ*t*), and the external resistance *R*_ext_ is much larger than the total device resistance, then the measured d.c. voltage *V*_c_(Δ*t*) is approximately equal to (*R*_j_/*T*_R_)∫*I*_2_(*t*, Δ*t*)d*t*, where *T*_R_ is the pulse repetition period, *R*_j_ is the resistance of the metal-MoS_2_ junction, and the time integral is over one complete period. As the time delay Δ*t* becomes much longer than the duration of *I*_1_(*t*), one expects *V*_c_(Δ*t*) to approach (2*R*_j_/*T*_R_)∫*I*_1_(*t*)d*t*.

### Experimental results

**Figure 2:**
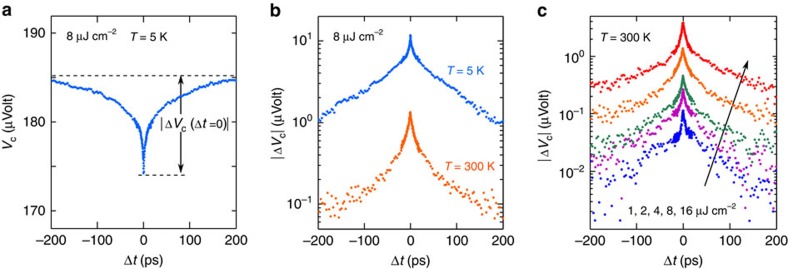
TPPC experiment results. (**a**) The measured two-pulse photovoltage correlation (TPPC) signal *V*_c_(Δ*t*) is plotted as a function of the time delay Δ*t* between the pulses. *T*=5 K and gate bias is 0 V. The pump fluence is 8 μJ cm^−2^. The quantity Δ*V*_c_(Δ*t*) is the difference between *V*_c_(Δ*t*) and its maximum value which occurs when Δ*t*→∞. (**b**) |Δ*V*_c_(Δ*t*)| is plotted on a log scale as a function of the time delay Δ*t* between the pulses to show the two distinct timescales exhibited by *V*_c_(Δ*t*). The two curves are for two different extreme temperatures: *T*=5 K and *T*=300 K. The plot shows two distinct timescales: (i) a fast timescale of ∼4.3 ps, and (ii) a slow timescale of ∼105 ps. Gate bias is 0 V. The pump fluence is 8 μJ cm^−2^. The two different timescales are observed at both temperatures and these timescales are largely temperature independent. (**c**) The measured TPPC signal |Δ*V*_c_(Δ*t*)| is plotted as a function of the time delay Δ*t* between the pulses for different pulse fluences: 1, 2, 4, 8 and 16 μJ cm^−2^. *T*=300 K and gate bias is 0 V. The two different timescales are observed at all values of the pump fluence and these timescales are not very sensitive to the pump fluence.

Figure 2a shows the measured two-pulse photovoltage correlation signal *V*_c_(Δ*t*) plotted as a function of the time delay Δ*t* between the pulses. The substrate temperature is 5 K, the gate bias is 0 V, and the pump fluence is 8 μJ cm^−2^. *V*_c_(Δ*t*) is minimum when the two pulses completely overlap in time (that is, when Δ*t*=0). This implies, not surprisingly, that the photovoltage response of the detector to an optical pulse is a sublinear function of the optical pulse energy. As Δ*t* increases from zero, *V*_c_(Δ*t*) also increases from its minimum value at Δ*t*=0. As Δ*t* becomes much longer than the duration of the response transient of the detector to an optical pulse, *V*_c_(Δ*t*) approaches a constant value. The timescales over which *V*_c_(Δ*t*) goes to the constant value are related to the timescales associated with the response transient of the detector to an optical pulse. These timescales are better observed in the measured data if the magnitude of Δ*V*_c_(Δ*t*), defined as *V*_c_(Δ*t*)−*V*_c_(∞), is plotted on a log scale, as shown in Fig. 2b. The plot in Fig. 2b shows two distinct timescales: (i) a fast timescale of ∼4.3 ps and (ii) a slow timescale of ∼105 ps. In different devices, the fast timescales were found to be in the 3–5 ps range, and the slow timescales were in the 90–110 ps range. The fast timescales imply (8 dB) current modulation bandwidths wider than 300 GHz.

Measurements were performed at different temperatures and using different pump pulse fluences to understand the mechanisms behind the photoresponse and the associated dynamics. Figure 2b shows |Δ*V*_c_(Δ*t*)| plotted for two different extreme temperatures: *T*=5 K and *T*=300 K. Gate bias is 0 V. The pump fluence is 8 μJ cm^−2^. Two distinct timescales are observed at both temperatures and these timescales are largely independent of the temperature. Measurements performed at intermediate temperatures provided the same results. |Δ*V*_c_(Δ*t*)| was found to be larger at smaller temperatures. We attribute this to the increase in the metal-semiconductor junction resistance *R*_j_ at lower temperatures. Figure 2c shows |Δ*V*_c_(Δ*t*)| plotted for different values of the pump pulse fluence. The pump pulse fluence was varied from 1 to 16 μJ cm^−2^. Higher values of the pump fluence were not used to avoid optical damage to the sample^12^. *T*=300 K and gate bias is 0 V. Figure 2c shows that the same two timescales are observed for all values of the pump fluence and these timescales are not very sensitive to the pump fluence. The observed timescales also did not change in any significant way under a positive or a negative gate bias (Supplementary Figs 3 and 4 and Supplementary Note 4). The internal and external detector quantum efficiencies were estimated from the measured values of *V*_c_(∞) and the junction resistance *R*_j_ to be in the 0.008–0.016 and 0.001–0.002 ranges, respectively.

The two different timescales observed in our two-pulse photovoltage correlation experiments match well with the two different timescales observed previously in ultrafast optical/THz pump–probe studies of carrier dynamics as well as in ultrafast photoluminescence studies of MoS_2_ monolayers^12^,^22^. It is therefore intriguing if the model for defect-assisted carrier recombination reported previously by the authors^12^,^28^ for explaining the ultrafast carrier dynamics in MoS_2_ monolayers can be used to obtain photovoltage correlations that are in good agreement with the experimental results reported in this letter. We show below that this is indeed the case.

### Ultrafast photoresponse of the metal-MoS_2_ junction

**Figure 3:**
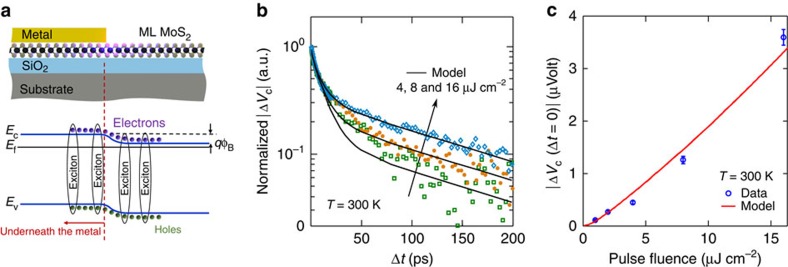
Theoretical modelling and fitting of TPPC experiment results. (**a**) The energy band diagram (bottom) of the the metal-MoS_2_ junction (top) is plotted as a function of the position in the plane of the MoS_2_ monolayer after photoexcitation with an optical pulse. The Schottky barrier height is *φ*_B_. The figure is not drawn to scale. (**b**) The measured (symbols) and computed (solid lines) photovoltage correlation signals |Δ*V*_c_(Δ*t*)|, normalized to the maximum value, are plotted as a function of the time delay Δ*t* for different pump fluence values: 4, 8 and 16 μJ cm^−2^. *T*=300 K. The carrier capture model reproduces all the timescales observed in the measurements over the entire range of the pump fluence values used. (**c**) The scaling of the measured (symbols) and computed (solid line) values of |Δ*V*_c_(Δ*t*=0)| with the pump pulse fluence is shown. The error bars are the recorded peak-to-peak noise level of lock-in amplifier during each measurement.

Understanding the ultrafast photoresponse of the detector, and in particular the short circuit current response *I*_2_(*t*, Δ*t*), is important for interpreting the experimental results. Figure 3a depicts the band diagram of the metal-MoS_2_ junction (plotted in the plane of the MoS_2_ layer) after photoexcitation with an optical pulse. Given the Schottky barrier height of 100–300 meV (ref. 8), the width of the MoS_2_ region near the metal with a non-zero lateral electric field is estimated to be to ∼100–300 nm (ref. 29). As a result of light diffraction from the edge of the metal contact, light scattering from the substrate, and plasmonic guidance, a portion of the MoS_2_ layer of length equal to a few hundred nanometres is photoexcited even underneath the metal (Supplementary Fig. 1 and Supplementary Note 1). The photoresponse of graphene photodetectors has been explained in terms of contributions from photovoltaic and photothermoelectric contributions^23^,^24^,^25^,^26^,^27^,^30^. In our MoS_2_ samples, the carrier mobilities and diffusivities are 2–3 orders of magnitude smaller than in graphene and the time period in which most of the photoexcited carriers recombine and/or are captured by defects is in the few picoseconds range^12^,^20^,^21^,^22^,^28^. Assuming similar mobilities and diffusivities of electrons and holes in MoS_2_, the photoexcited carriers, both free and bound (excitons), move, either by drift in the junction lateral electric field or by diffusion, less than ∼10 nm in 5 ps before they recombine and/or are captured by defects. The photoexcited carrier distributions therefore do not change significantly in space during their lifetime. Separation of electrons and holes either by the junction lateral electric field or at the metal-MoS_2_ interface will contribute to the integral ∫*I*_2_(*t*, Δ*t*)d*t*(∝*V*_c_(Δ*t*)) (the measured dependence of the photoresponse on the junction electric field and the gate voltage is discussed in the Supplementary Figs 3 and 4 and Supplementary Note 4. We assume that the integral ∫*I*_2_(*t*, Δ*t*)d*t* is approximately proportional to the integral ∫[*p*′(*t*, Δ*t*) + *n*′(*t*, Δ*t*)]d*t* (Supplementary Note 2). Here, *p*′(*t*, Δ*t*) and *n*′(*t*, Δ*t*) are the time-dependent photoexcited electron and hole densities in the junction, including carriers both free and bound (excitons). This assumption, although simple, allows one to relate the measured photoresponse to the carrier dynamics and, as shown below, the results thus obtained are in excellent agreement with the experiments. We expect that on much longer timescales, when the photoexcited carriers have recombined or been captured, the photoresponse is entirely thermoelectric in nature, as is the case in metal–graphene photodetectors^23^,^25^,^26^,^27^,^30^. But in this letter we focus on the dynamics occurring on only short timescales.

### Carrier capture and recombination model

It is known that most of the photoexcited carriers in monolayer MoS_2_ recombine nonradiatively^12^,^15^,^28^. The temperature independence as well as the sample dependence of the recombination rates in previously reported works suggested that free and bound (excitons) carriers recombine via capture by defects through Auger scattering^12^,^28^. Monolayer MoS_2_ can have several different kinds of defects, including grain boundaries, line defects, interstitials, dislocations and vacancies^31^,^32^,^33^,^34^,^35^,^36^,^37^. The strong electron and hole Coulomb interaction in monolayer TMDs makes defect capture via Auger scattering very effective^28^. The time constants observed in this work in the photovoltage correlations are also temperature independent (Fig. 2b) and also match well with the time constants observed previously in optical/THz pump–probe and photoluminescence measurements^12^,^22^. We therefore use the model for carrier capture by defects via Auger scattering in MoS_2_ presented by Wang *et al.*^12^,^28^ to model our TPPC experimental results (Supplementary Fig. 5 and Supplementary Note 5). The model assumes carrier capture by two different defect levels, one fast and one slow^12^. The essential dynamics captured by the model, and their relationships to the experimental observations, are as follows^12^. After photoexcitation, electrons and holes thermalize and lose most of their energy on a timescale much shorter than our experimental resolution (∼0.5–1.0 ps) and, therefore, thermalization is assumed to happen instantly in our model^38^. Most of the photoexcited holes (both free and bound), followed by most of the electrons, are captured by the fast defects within the first few picoseconds after photoexcitation. During the same period, a small fraction of the holes is also captured by the slow defects. This rapid capture of the photoexcited electrons and holes is responsible for the fast time constant observed in our photovoltage correlation experiments. The remaining photoexcited electrons are captured by the slow defects on a timescale of 65–80 ps and this slow capture of electrons is responsible for the slow time constant observed in our photovoltage correlation experiments. We should point out here that the two time constants observed in the photovoltage correlation signal Δ*V*_c_(Δ*t*) are always slightly longer than the corresponding two time constants exhibited by the carrier densities in direct optical pump–probe and measurements^12^, as is to be expected in the case of correlation measurements. Finally, the superlinear dependence of the carrier capture rates on the photoexcited carrier densities in the model, and therefore on the optical pulse energy, results in the experimentally observed negative value of the photovoltage correlation signal Δ*V*_c_(Δ*t*=0) at zero time delay. The values of the fitting parameters used in the theoretical model to fit the experimental data are given in the Supplementary Table 1 and are almost identical to the values extracted previously from direct optical pump–probe measurements of the carrier dynamics in monolayer MoS_2_ (ref. 12).

The comparison between the data and the model are shown in Fig. 3b which plots the measured and computed photovoltage correlation |Δ*V*_c_(Δ*t*)| as a function of the time delay Δ*t* for different pump fluence values: 4, 8 and 16 μJ cm^−2^. All curves are normalized to a maximum value of unity (since the model gives the photovoltage correlation signal up to a multiplicative constant). The model not only reproduces the very different timescales observed in |Δ*V*_c_(Δ*t*)| measurements, it achieves a very good agreement with the data over the entire range of the pump fluence values used in our experiments for the same values of the parameters (Supplementary Table 1). Figure 3c shows the scaling of the measured and computed values of |Δ*V*_c_(Δ*t*=0)| with the pump pulse fluence. Again, a very good agreement is observed between the model and the data.

## Discussion

Our results reveal the fast response time and the wide bandwidth of metal-MoS_2_ photodetectors and show that these detectors can be used for ultrafast applications. Our results also shed light on the carrier recombination mechanisms and the associated timescales. Although we focused mainly on the carrier dynamics in this paper, the device intrinsic resistances and capacitances are not expected to fundamentally limit the device speed because of the rather small capacitances associated with the lateral metal-semiconductor junctions (Supplementary Note 3). An obstacle to using TMDs in practical light emission and detection applications is the small values of the reported quantum efficiencies in these materials^5^,^6^,^7^,^8^,^9^,^12^,^13^,^14^,^15^. In photodetectors, the response speed and the quantum efficiency are often inversely related^39^. In most semiconductor photovoltaic detectors with large carrier mobilities and long recombination times (for example, group III–V semiconductor photodetectors^39^), the transit time of the photogenerated carriers through the junction depletion region determines the detector bandwidth^39^. Given the relatively small carrier mobilities and diffusivities in MoS_2_, the fast carrier recombination times determine the speed in our metal-MoS_2_ detectors. The price paid for the the fast response time is the small internal quantum efficiency: most of the photogenerated carriers recombine before they make it out into the circuit. The best reported carrier mobilities in monolayer MoS_2_ are an order of magnitude larger than in our devices and, therefore, metal-MoS_2_ photodetectors with internal quantum efficiencies around 0.1 (approximately an order of magnitude larger than of our devices) are possible without sacrificing the wide bandwidth. Density of defects, which contribute to carrier trapping and recombination, is also a parameter that can be potentially controlled in 2D TMD optoelectronic devices to meet the requirements for ultrafast or high quantum efficiency applications. In addition, vertical heterostructures of 2D TMD materials can also be used to circumvent the transport bottleneck in high speed applications 40.

## Methods

### Device fabrication and characterization

Monolayer MoS_2_ samples were mechanically exfoliated from bulk MoS_2_ crystal (obtained from 2D Semiconductors) and transferred onto highly *n*-doped Si substrates with 90 nm thermal oxide. Monolayer sample thickness was confirmed by Raman and reflection spectroscopies^41^. Au metal contacts (with a very thin Cr adhesion layer) were patterned and deposited onto the samples using electron-beam lithography. The doped Si substrate acted as the back gate. Microscope image of a 10 × 10 μm^2^ area device is shown in Fig. 1a. Carrier densities and mobilities in the devices were determined using electrical transport measurements on devices of different dimensions. The devices were found to be *n*-doped with electron densities around 1 × 10^12^–2 × 10^12^ cm^−2^ (under zero gate bias). The intrinsic doping was attributed to impurities and defect levels^8^. The electron mobility in the devices was found to be in the 15–20 cm^2^ V^−1^ s^−1^ range at 5 K. The zero gate bias device resistance was typically <1 MΩ at all temperatures for a 10 × 10 μm^2^ area device. While the device resistance decreased under a positive gate bias, no signature of hole conduction was observed even when a large negative gate bias was applied indicating that the Fermi level in the MoS_2_ layer was likely pinned at defect levels within the bandgap under a negative gate bias. The devices were mounted in a helium-flow cryostat and the temperature was varied between 5 and 300 K during measurements. The zero gate bias device resistance was found to be a function of the temperature and decreased almost linearly with the temperature from ∼1 MΩ at 5 K to values 5–7 times smaller at 300 K. The total device resistance was dominated by the metal Schottky contacts to the device. For example, at 5 K the resistance contributed by the 10 × 10 μm^2^ area MoS_2_ strip is estimated to be in the 0.10–0.20 MΩ range (from the measured mobility values), which is approximately only one-tenth to one-fifth of the total device resistance. The reported Schottky barrier heights between similarly *n*-doped monolayer MoS_2_ and Au/Cr contacts are in the 100–300 meV range^8^. Depending on how strongly the Fermi level gets pinned at the defect levels in the bandgap, the lateral potential drop in the MoS_2_ layer at the metal-MoS_2_ interface (depicted in Fig. 3a) could be equal to or smaller than the Schottky barrier height.

### TPPC experiment setup

In the TPPC measurements, the two 452 nm optical pulses were cross-polarized to minimize interference and focused onto the device using a 20 × or a 100 × microscope objective resulting in a minimum focus spot size of around 0.75 μm. Optical absorption in monolayer MoS_2_ layers was characterized using a confocal microscope-based relection/transmission setup^12^ and yielded ∼11–12% absorption (single-pass) in monolayer MoS_2_ on oxide at 452 nm (pump pulse wavelength). Measurement of the photovoltage using a high input impedance voltage amplifier (Lock-in in our case) was found to give a much better signal-to-noise ratio than the measurement of the photocurrent directly using a low input impedance transimpedance amplifier.

## Additional information

**How to cite this article:** Wang, H. *et al.* Ultrafast response of monolayer molybdenum disulfide photodetectors. *Nat. Commun.* 6:8831 doi: 10.1038/ncomms9831 (2015).

## Supplementary Material

Supplementary Information Supplementary Figures 1-5, Supplementary Table 1, Supplementary Notes 1-5 and Supplementary References
