# Supplementary material for: Ultrafast response of monolayer molybdenum disulfide photodetectors
Source: Nat Commun. 2015 Nov 17;6:8831. doi: 10.1038/ncomms9831 (PMC4660040; doi:10.1038/ncomms9831)
Supplement: Supplementary Information — Supplementary Figures 1-5, Supplementary Table 1, Supplementary Notes 1-5 and Supplementary References [file ncomms9831-s1.pdf]

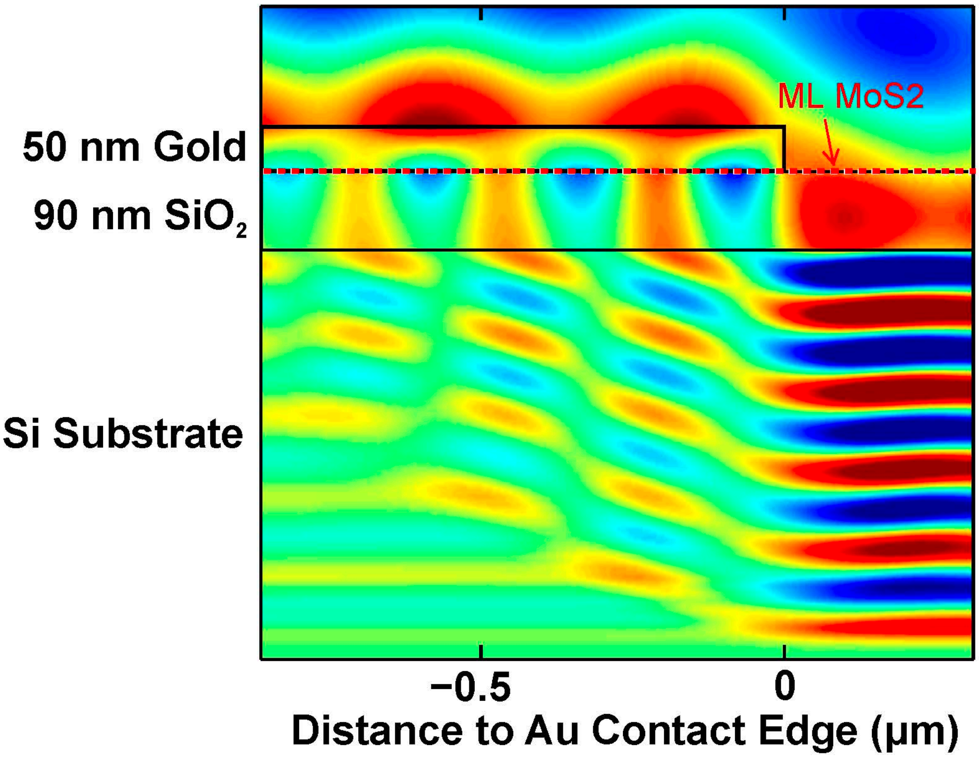

Supplementary Figure 1: **Numerical simulation of photoexcitation at metal-MoS_2 junction.** Finite Difference Time domain (FDTD) simulation of the optical excitation of the metal-MoS_2 junction is shown. The location of the MoS_2 layer is indicated by the dashed line. The simulation shows that a portion of the MoS_2 layer of length equal to a few hundred nanometers is photoexcited even underneath the metal.

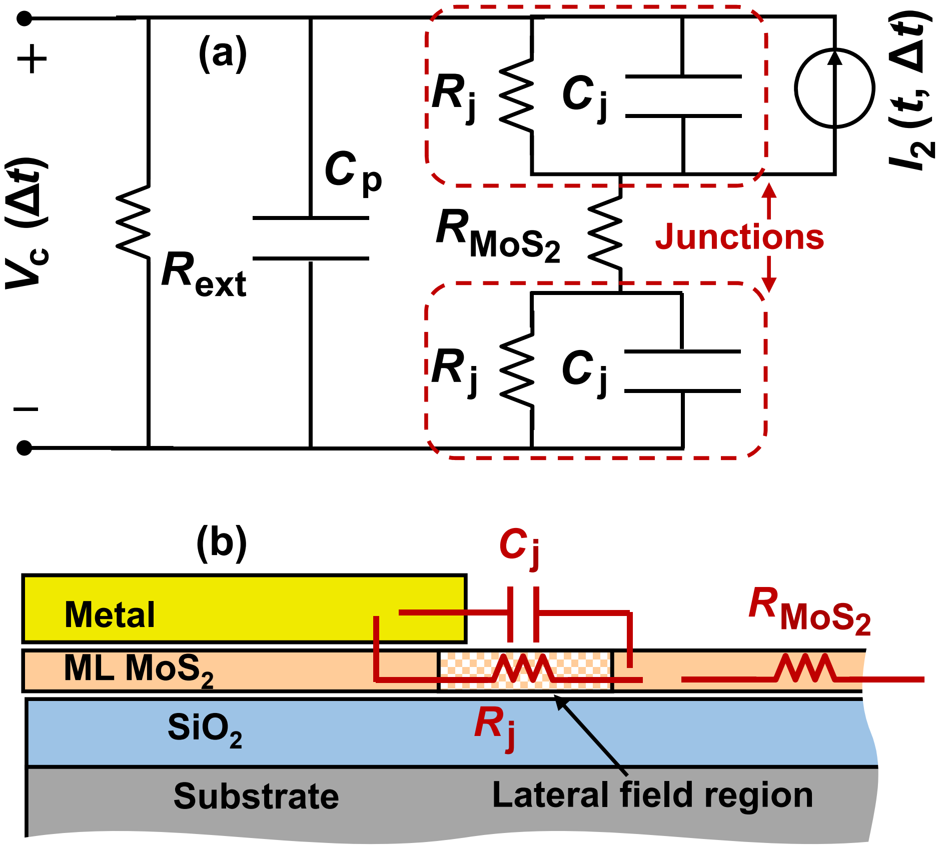

Supplementary Figure 2: Circuit model of metal-MoS_2 photodetector. A high-frequency circuit model of the photodetector is shown in (a). The resistances and capacitances associated with the metal-MoS_2 junction are depicted in (b). R_j and C_j are the resistance and the capacitance associated with the metal-MoS_2 junction. $R_{\mathrm{MoS_2}}$ is the resistance of the undepleted MoS_2 region. C_p is an external parasitic capacitance and R_ext is the external circuit resistance (including the input resistance of the measurement instrument).

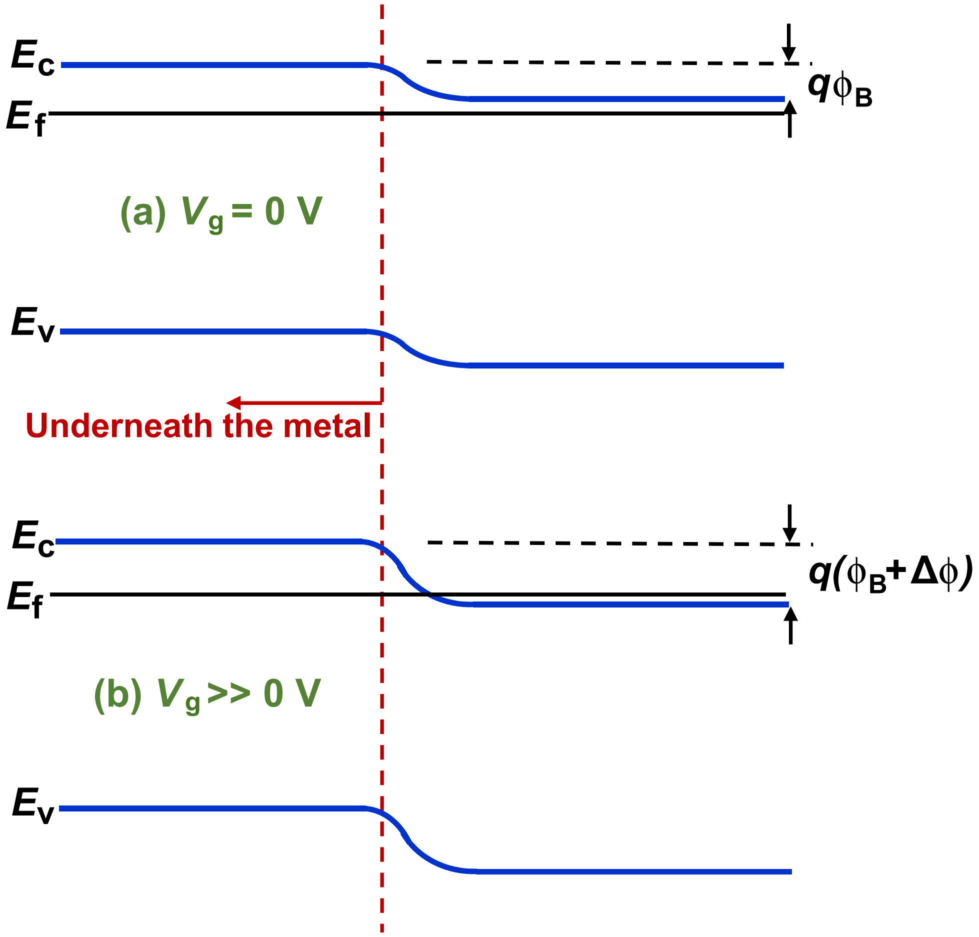

Supplementary Figure 3: **Band diagram of metal-MoS$_2$ junction.** The effect of the gate voltage V_{g} on the energy band diagram of the the metal-MoS$_2$ junction is depicted when (a) $V_{\mathrm{g}} = 0$ V, and (b) $V_{\mathrm{g}} >> 0$ V. The in-plane electric field increases near the metal junction with an increase in the gate voltage.

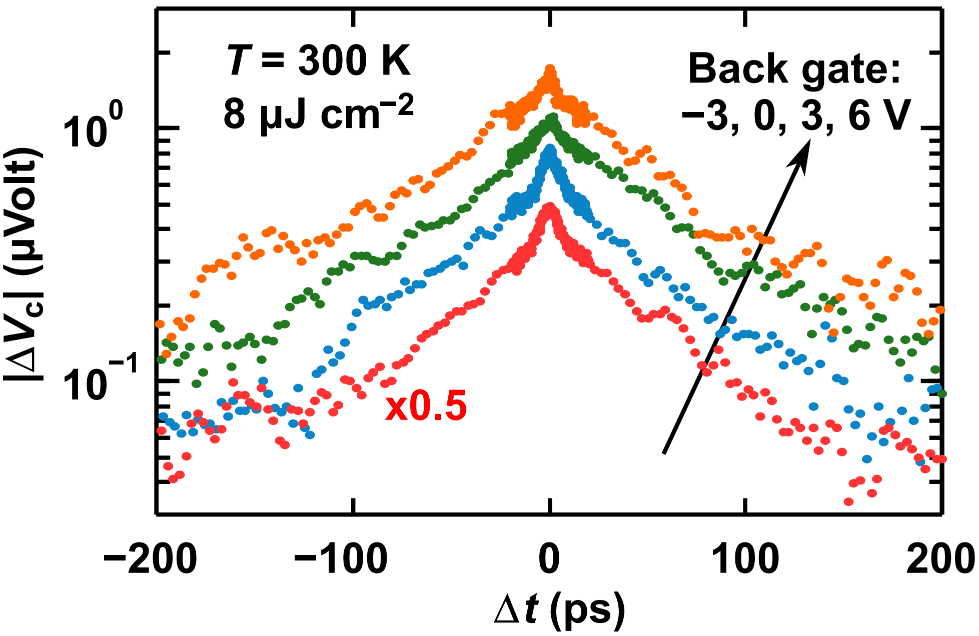

Supplementary Figure 4: **Gated TPPC experiment results.** The measured two-pulse photovoltage correlation (TPPC) signal $|\Delta V_{\mathrm{c}}(\Delta t)|$ is plotted as a function of the time delay Δt between the pulses for different gate bias values: -3, 0, 3, 6 V. $T = 300$K. The pump fluence is 8 μJ cm^{-2}. As in Figure 2(b,c) in the article, two distinct time scales are observed in the dynamics and these time scales are largely independent of the gate bias.

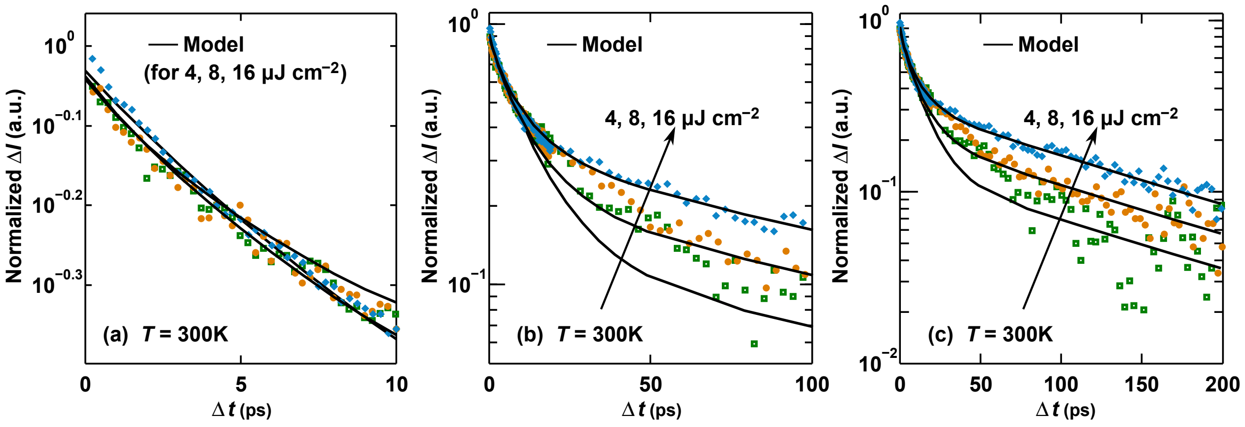

Supplementary Figure 5: **Details of the theoretical fitting.** The measured (symbols) and computed (solid lines) photovoltage correlation signals $|\Delta V_{\mathrm{c}}(\Delta t)|$, normalized to the maximum value, are plotted as a function of the time delay Δt for different pump fluence values: 4, 8, and 16 μJ cm^{-2} and for different temporal resolutions (a-c). T = 300K. Note that at short time scales the measured transients are not exactly decaying exponentials. The carrier capture model reproduces all the time scales observed in the measurements over the entire range of the pump fluence values used.

$B_{\mathrm{f}} n_{\mathrm{f}}$	0.73 ± 0.05 cm^2 s^{-1}
$B_{\mathrm{s}} n_{\mathrm{s}}$	$2.79 \pm 1 \times 10^{-2}$ cm^2 s^{-1}
A_{s}	$9.5 \pm 1 \times 10^{-15}$ cm^4 s^{-1}
A_{f}	$(1.0 \pm 0.2) B_{\mathrm{f}}$
$n_{\mathrm{f/s}}$	5.0×10^{12} cm^{-2}
n_{o}	8×10^{11} cm^{-2}

Supplementary Table I: **Fitting parameters.** Parameter values used in the simulations to fit the photovoltage correlation data.

Supplementary Note 1: Numerical Simulation of the Photoexcitation of the Metal-$\mathbf{MoS_2}$ Junction

A Finite Difference Time domain (FDTD) simulation of the optical excitation of the metal-MoS_2 junction by normally incident radiation is shown in Supplementary Figure 1. The location of the MoS_2 layer is indicated by the dashed line. The simulation shows that as a result of light diffraction from the edge of the metal contact, light scattering from the substrate, and plasmonic guidance, a portion of the MoS_2 layer of length equal to a few hundred nanometers is photoexcited even underneath the metal.

Supplementary Note 2: Details on the Ultrafast Photoresponse of the Metal-$\mathbf{MoS_2}$ Junction

The nature of the ultrafast photoresponse of the metal-MoS_2 junction is discussed here. Figure 3(a) in the article depicts the band diagram of the metal-MoS_2 junction (plotted in the plane of the MoS_2 layer) after photoexcitation with an optical pulse. Given the Schottky barrier height of 100-300 meV [1], the width of the MoS_2 region near the metal with a non-zero lateral electric field is estimated to be to ~100-300 nm [2]. Note that the lateral electric field right underneath the metal is expected to be very small. As a result of light diffraction from the edge of the metal contact, light scattering from the substrate, and plasmonic guidance, a portion of the MoS_2 layer of length equal to a few hundred nanometers is photoexcited even underneath the metal (see the discussion above). Assuming similar mobilities and diffusivities of electrons and holes in MoS_2, the photoexcited carriers, both free and bound (excitons), move, either by drift in the junction lateral electric field or by diffusion, less than ~10 nm in 5 ps before they recombine and/or are captured by defects. The photoexcited carrier distributions therefore do not change significantly in space during their lifetime.

The ultrafast current response $I_2(t, \Delta t)$ of a short circuited junction in response to two time-delayed optical pulses is expected to be fairly complicated. In TPPC experiments the quantity measured is the time integral $\int I_2(t, \Delta t)\, dt\, (\propto V_c(\Delta t))$. The motion of the photoexcited electrons and holes in a short circuited junction causes capacitive (i.e. displacement) currents in the external circuit in order to keep the potential across the shorted junction from changing in accordance with the Ramo-Shockley theorem [3, 4]. However, if the photoexcited carriers recombine before they make it out into the circuit then the net contribution of the capacitive currents to the integral $\int I_2(t, \Delta t)\, dt$ is identically zero.

Photoexcited electrons and holes can be separated before they form excitons by the lateral electric field in the junction and this constitutes the standard drift current contribution to the

detector short circuit current response $\int I_2(t, \Delta t)\, dt$. A photoexcited electron and a hole (free or belonging to an exciton) in the MoS_2 layer underneath the metal can also be separated at the metal-MoS_2 heterojunction. The hole can tunnel into the metal leaving behind the electron which is then swept by the lateral electric field to the opposite side of the junction. The electron can also tunnel into the metal leaving behind the hole which will then have a difficult time traversing the lateral field region (moving against the electric field) and making it to the opposite side of the junction. This argument shows that even if the probabilities of the electron and the hole tunneling into the metal are similar, the lateral field in the junction ensures that the process in which the hole tunnels into the metal makes the dominant contribution to the short circuit current response $\int I_2(t, \Delta t)\, dt$. The experimentally measured sign of the photovoltage, and the photocurrent (see Figure 1(b) in the article), agrees with the above arguments.

The discussion above shows that the short circuit current response $\int I_2(t, \Delta t)\, dt$ is proportional to the junction lateral electric field strength, and to the time integral of the photoexcited free electron and hole densities as well as to the time integral of the bound (exciton) electron and hole densities. Assuming similar electron and hole mobilities, one may write,

$$\int I_2(t, \Delta t)\, dt \propto k_{\mathrm{f}} \int \left[p'_{\mathrm{f}}(t, \Delta t) + n'_{\mathrm{f}}(t, \Delta t) \right] \, dt + k_{\mathrm{b}} \int \left[p'_{\mathrm{b}}(t, \Delta t) + n'_{\mathrm{b}}(t, \Delta t) \right] \, dt \tag{1}$$

Here, $n'_{\mathrm{f/b}}(t, \Delta t)$ and $p'_{\mathrm{f/b}}(t, \Delta t)$ are the spatially-averaged free/bound (f/b) photoexcited electron and hole densities in the junction, respectively. Since photoexcited electrons and holes don't have time to move much before they recombine and/or are captured by defects, spatial dynamics of the carrier densities are not important. The constants k_{f} and k_{b} capture the difference in the relative contributions from free and bound carriers to the current response. If one assumes that $k_{\mathrm{f}} \approx k_{\mathrm{b}}$ then,

$$\int I_2(t, \Delta t)\, dt \propto \int \left[p'(t, \Delta t) + n'(t, \Delta t) \right] \, dt \tag{2}$$

where $n'(t, \Delta t)$ and $p'(t, \Delta t)$ are the total photoexcited electron and hole densities in the junction, respectively, including carriers both free and bound (excitons). The assumption $k_{\mathrm{f}} \approx k_{\mathrm{b}}$ will hold if the short circuit current is dominated by the free and bound electrons and holes that get separated at the metal-MoS_2 heterojunction. Since the junction resistance R_{j} is expected to be largely determined by the transport across the metal-MoS_2 heterojunction rather than by the transport across the MoS_2 region, the assumption $k_{\mathrm{f}} \approx k_{\mathrm{b}}$ is a decent approximation if not an excellent one.

Supplementary Note 3: High Frequency and Low Frequency Circuit Models of the Metal-MoS$_2$ Junction

A circuit model of the photodetector is shown in Supplementary Figure 2(a) and the resistances and capacitances associated with the metal-MoS$_2$ junction are shown in Supplementary Figure 2(b). The time-dependent short circuit current response of the photodetector to two optical pulses separated by time Δt is $I_2(t, \Delta t)$. The short circuit current response $I_2(t, \Delta t)$ represents the photocurrent measured if the illuminated junction were shorted. In our experiments, the quantity measured is the DC value $V_{\mathrm{c}}(\Delta t)$ of the open circuit voltage. Assuming periodic excitation of the device with the optical pulses, $V_{\mathrm{c}}(\Delta t)$ can be written as,

$$V_{\mathrm{c}}(\Delta t) = \frac{1}{T_{\mathrm{R}}} \frac{R_{\mathrm{j}} R_{\mathrm{ext}}}{R_{\mathrm{d}} + R_{\mathrm{ext}}} \int I_2(t, \Delta t) dt \approx \frac{R_{\mathrm{j}}}{T_{\mathrm{R}}} \int I_2(t, \Delta t) dt \tag{3}$$

Here, the total device resistance R_{d} equals $2R_{\mathrm{j}} + R_{\mathrm{MoS_2}}$, T_{R} is the period of the optical excitation, and the time integrals above are performed over one complete period. The approximate equality above follows from the fact that in our experiments, $R_{\mathrm{ex}} >> R_{\mathrm{d}}$. Note that all the capacitances drop out in the expression for $V_{\mathrm{c}}(\Delta t)$. Therefore, one can use the low-frequency circuit model shown in Figure 1(c)(in the article) when calculating $V_{\mathrm{c}}(\Delta t)$.

It is instructful to determine whether the intrinsic device resistances and capacitances could fundamentally limit the high speed performance. The frequency dependent small-signal open circuit voltage response and short circuit current response of the detector can also be evaluated using the circuit shown in Figure 2(a) under the assumption that $I_2(t) = \mathrm{R}e\left\{i_2(\omega)e^{-i\omega t}\right\}$. The open circuit voltage response is (assuming $R_{\mathrm{ext}} = \infty$),

$$\frac{v_{\mathrm{c}}(\omega)}{i_2(\omega)} = \frac{R_{\mathrm{j}}}{1 - i\omega C_{\mathrm{j}} R_{\mathrm{j}} - i\omega C_{\mathrm{p}}(2R_{\mathrm{j}} + R_{\mathrm{MoS_2}}) + (i\omega)^2 C_{\mathrm{p}} C_{\mathrm{j}} R_{\mathrm{j}} R_{\mathrm{MoS_2}}} \tag{4}$$

If one ignores the parasitic capacitance C_{p} then the circuit bandwidth is set by the time constant $R_{\mathrm{j}} C_{\mathrm{j}}$. The junction resistance R_{j} is dominated by the metal-semcionductor contact resistance. In the case of MoS$_2$, the contact resistance values are in the 1-10 kΩ-μm range at room temperature [8]. Because of the 2D nature of the metal-semiconductor junction, the junction capacitance C_{j} is very small and entirely due to the fringing fields. For a $\sim$50 nm thick metal contact layer, C_{j} is estimated to be less than .03 fF/μm [2]. Therefore, the relevant time constant is estimated to be shorter than a picosecond. The short circuit current response is (assuming $R_{\mathrm{ext}} = 0$),

$$\frac{i_{\mathrm{ext}}(\omega)}{i_2(\omega)} = \frac{R_{\mathrm{j}}}{2R_{\mathrm{j}} + R_{\mathrm{MoS_2}} - i\omega C_{\mathrm{j}} R_{\mathrm{j}} R_{\mathrm{MoS_2}}} \tag{5}$$

In this case, the circuit bandwidth is set by the time constant $C_{\mathrm{j}} R_{\mathrm{j}} R_{\mathrm{MoS_2}}/(2R_{\mathrm{j}} + R_{\mathrm{MoS_2}})$. If $R_{\mathrm{j}} << R_{\mathrm{MoS_2}}$, as would be the case if the doping in the MoS$_2$ sheet is small, then the time

constant equals $\sim R_{\rm j}C_{\rm j}$, which is the same as for the open circuit voltage response. If on the other hand $R_{\rm j} >> R_{\rm MoS_2}$, then the time constant equals $\sim 0.5 R_{\rm MoS_2} C_{\rm j}$. Assuming an electron doping of $\sim 2 \times 10^{12}$ cm^{-2} (as in our devices) and a modest electron mobility of $\sim$20 cm^2/V-s (as in our devices), and a device length of 1 μm, the value of $R_{\rm MoS_2}$ comes out to be $\sim$150 kΩ-μm and the time constant comes out to be $\sim$2.25 ps. Finally, the capacitance $C_{\rm p}$ could come from the fringing fields between the two metal contacts and therefore its effect on the open circuit voltage response ought to be considered. For example, consider $\sim$50 nm thick metal contact layers that are one micron apart. The capacitance $C_{\rm p}$ is estimated to be less than .02 fF/μm [9]. The relevant time constant is $C_{\rm p}(2R_{\rm j} + R_{\rm MoS_2})$ and, assuming $R_{\rm j} << R_{\rm MoS_2}$, the time constant is found to be $\sim$3.0 ps. Therefore, in all the cases the intrinsic device resistances and capacitances are not expected to fundamentally limit the speed of operation of the detectors considered in this work.

Supplementary Note 4: Gate Bias Dependence of the Photoresponse

The effect of the gate voltage on the photoresponse and the TPPC measurements is discussed here. Increasing the back gate voltage increases the electron density in the MoS_2 layer and raises the Fermi level with respect to the conduction band edge. However, in the region of the MoS_2 layer which is underneath the metal the electron density as well as the position of the Fermi level with respect to the conduction band edge remains unchanged. This is because the metal on top screens all the extra charges on the gate as the gate voltage is increased. Consequently, the in-plane lateral electric field in the MoS_2 layer increases near the metal junction with an increase in the gate voltage, as depicted in Supplementary Figure 3. The exact magnitude of the increase in the in-plane electric field with the gate voltage is hard to predict since the result could depend on the degree of Fermi level pinning on defect states within the bandgap in MoS_2. Nevertheless, one would expect the measured photoresponse to also increase with the gate voltage since, as argued in this paper, the photoresponse is proportional to the in-plane electric field [5].

Supplementary Figure 4 shows the measured $|\Delta V_{\rm c}(\Delta t)|$ plotted as a function of the time delay Δt between the pulses for different gate bias values: -3, 0, 3, 6 V. T =300K. The pump fluence is 8 μJ cm^{-2}. As in Figure 2(b,c) in the article, two distinct time scales are observed in the dynamics and, within the accuracy of our measurements, these time scales are largely independent of the gate bias. As expected from our model, the overall signal level increases with the gate bias.

Supplementary Note 5: Theoretical Model for Carrier Capture and Recombination via Auger Scattering

We use the model for carrier capture by defects via Auger scattering in MoS_2 [6, 7] to model our experimental TPPC results. The model assumes carrier capture by two different defect levels, one

fast (f) and one slow (s). Keeping only the dominant Auger capture processes in an n-doped sample, and ignoring carrier emission processes for simplicity, the rate equations for the carrier densities and defect occupation probabilities with photoexcitation by two time-delayed optical pulses can be written as follows [6],

$$\frac{dn(t,\Delta t)}{dt} = -A_{\rm f}n_{\rm f}(1-F_{\rm f})n^2 - A_{\rm s}n_{\rm s}(1-F_{\rm s})n^2 + gI_{\rm p}(t) + gI_{\rm p}(t-\Delta t) \quad (6)$$

$$\frac{dp(t,\Delta t)}{dt} = -B_{\rm f}n_{\rm f}F_{\rm f}np - B_{\rm s}n_{\rm s}F_{\rm s}np + gI(t) + gI(t-\Delta t) \quad (7)$$

$$n_{\rm f/s}\frac{dF_{\rm f/s}(t,\Delta t)}{dt} = A_{\rm f/s}n_{\rm f/s}n^2(1-F_{\rm f/s}) - B_{\rm f/s}n_{\rm f/s}npF_{\rm f/s} \quad (8)$$

Here, $n(t,\Delta t)$ and $p(t,\Delta t)$ are the total electron and hole densities, respectively, including both free and bound (excitons) carriers and $n(t,\Delta t) = n_{\rm o} + n'(t,\Delta t)$, where $n_{\rm o}$ is the doping density. $F_{\rm f/s}$ are the defect occupation probabilities. $A_{\rm f/s}$ and $B_{\rm f/s}$ are the Auger capture rate constants for electrons and holes, respectively. $n_{\rm f/s}$ are the defect densities. $I_{\rm p}(t)$ is the optical pulse intensity (μW cm^{-2}) and g, determined from the measured MoS_2 optical absorption at the wavelength of the optical pulse excitation, equals $\sim$2.5$\times$10^{11} (μJ)$^{-1}$ and corresponds to around 11% absorption in monolayer MoS_2 on oxide at 452 nm wavelength [6]. In our n-doped sample, the defects are assumed to be fully occupied before photoexcitation. The above rate equations can be solved in time and the resulting photoexcited carrier densities integrated in time to yield the measured photovoltage correlation signal (up to a multiplicative constant). The results are shown in Supplementary Figure 5(a-c) and compared with the measurement results.

The values of the fitting parameters used in the theoretical model to fit the experimental data (see Figure 3 in the article) are listed in Supplementary Table 1. These values are almost identical to the values extracted from direct optical pump-probe measurements of the carrier dynamics in monolayer MoS_2 [6]. The value of the doping density, $n_{\rm o} \sim 8\times10^{11}$ cm^{-2}, needed to obtain a good match with the experiments is much smaller than the doping density determined from electrical transport measurements. This difference is attributed to the fact that the carrier density in MoS_2 near the metal contact is indeed much smaller than in the bulk of the device (see the energy band diagram in Figure 3(a) in the article).

In the simulations, we assumed, for simplicity, that the defect occupation probability before photoexcitation is unity. This assumption might not always hold. The defect occupation probability could be a function of the temperature or the gate bias and this could have observable consequences. If the occupation probability of the slow defects before photoexcitation is smaller at higher temperatures, which is plausible, then the ratio of $|\Delta V_{\rm c}(\Delta t = 0)|$ to $|\Delta V_{\rm c}(\Delta t)|$, when

Δt is at the boundary between the slow and fast time constant regions, will be larger at higher temperatures since fewer photoexcited holes would have been captured by the slow defects during the initial fast transient and, consequently, fewer photoexcited electrons would have remained in the conduction band after the fast transient is over. This could explain the small decrease in the ratio of $|\Delta V_c(\Delta t = 0)|$ to $|\Delta V_c(\Delta t)|$ at the boundary between the slow and fast time constant regions observed in our measurements at the lower temperature in Figure 2(b) in the article. Note also that the ratio of $|\Delta V_c(\Delta t = 0)|$ to $|\Delta V_c(\Delta t)|$, when Δt is at the boundary between the slow and fast time constant regions, is also smaller at more positive gate bias values (see Supplementary Figure 4). This trend is similar to the trend observed in $|\Delta V_c(\Delta t)|$ when going to lower substrate temperatures, and, as before, we attribute this to a larger initial occupancy of the slow defect states just before photoexcitation at more positive gate bias values.

SUPPLEMENTARY REFERENCES

[1] Sundaram, R. S.; Engel, M.; Lombardo, A.; Krupke, R.; Ferrari, A. C.; Avouris, P.; Steiner, M. Electroluminescence in single layer MoS_2. *Nano Letters*, *13*, 1416–1421 (2013)

[2] S.G. Petrosyan, A. Y. S. Contact phenomena in low-dimensional electron systems. *Sov. Phys. JETP*, *69*, 1261–1266 (1989)

[3] Shockley, W. Currents to conductors induced by a moving point charge. *Journal of Appl. Phys.*, *9*, 635–636 (1938)

[4] Ramo, S. Currents induced by electron motion. *Proceedings of the IRE*, *27*, 584–585, (1939)

[5] Lopez-Sanchez, O.; Lembke, D.; Kayci, M.; Radenovic, A.; Kis, A. Ultrasensitive photodetectors based on monolayer MoS_2. *Nature Nanotech.*, *8*, 497–501 (2013)

[6] Wang, H.; Zhang, C.; Rana, F. Ultrafast dynamics of Defect-Assisted Electron-Hole recombination in monolayer MoS_2. *Nano Lett.*, *15*, 339–345 (2015)

[7] Wang, H.; Strait, J. H.; Zhang, C.; Chen, W.; Manolatou, C.; Tiwari, S.; Rana, F. Fast exciton annihilation by capture of electrons or holes by defects via Auger scattering in monolayer metal dichalcogenides. *Phys. Rev. B*, *91*, 165411 (2015)

[8] Kappera, R.; Voiry, D.; Yalcin, S. E.; Branch, B.; Gupta, G.; Mohite, A. D.; Chhowalla, M. Phase-engineered low-resistance contacts for ultrathin MoS2 transistors. *Nature Materials*, *13*, 1128–1134 (2014)

[9] Garg, R,; Inder, B.; Bozzi, M.; *Microstrip Lines and Slotlines*, Artech House (2013)
